# Immune characterization of the HBHA-specific response in Mycobacterium tuberculosis-infected patients with or without HIV infection

**DOI:** 10.1371/journal.pone.0183846

**Published:** 2017-08-24

**Authors:** Teresa Chiacchio, Giovanni Delogu, Valentina Vanini, Gilda Cuzzi, Flavio De Maio, Carmela Pinnetti, Alessandro Sampaolesi, Andrea Antinori, Delia Goletti

**Affiliations:** 1 Translational Research Unit, Department of Epidemiology and Preclinical Research, “L. Spallanzani” National Institute for Infectious Diseases (INMI), Rome, Italy; 2 Institute of Microbiology, Università Cattolica del Sacro Cuore – Fondazione Policlinico Universitario Gemelli, Rome, Italy; 3 Department of Clinical and Clinical Research, “L. Spallanzani” National Institute for Infectious Diseases (INMI), IRCCS, Rome, Italy; Universita degli Studi di Palermo, ITALY

## Abstract

**Introduction:**

RD1-based Interferon-γ Release Assays (IGRAs) cannot distinguish latent from active tuberculosis (TB) disease. Conversely, a positive response to heparin-binding haemagglutinin (HBHA)-based IGRAs, among TB-infected subjects, correlates with *Mycobacterium tuberculosis* (*Mtb)* containment and low risk of TB progression. The aim of this study was to characterize HBHA-immune responses in HIV-infected and uninfected subjects with active TB or latent TB infection (LTBI).

**Methods:**

49 subjects were prospectively enrolled: 22 HIV-uninfected (13 TB, 9 LTBI) and 27 HIV-infected (12 HIV-TB, 15 HIV-LTBI). Whole blood and peripheral blood mononuclear cells were stimulated with HBHA and RD1 antigens. Interferon (IFN)γ release was evaluated by ELISA whereas cytokine profile [IFNγ, tumor necrosis (TNF)α, interleukin (IL)2] and phenotype (CD45RA, CCR7) by flow cytometry.

**Results:**

Among LTBI individuals, HBHA stimulation induced IFNγ release in all the HIV-uninfected, while, only 4/15 HIV-infected responded. Within the active TB, only 5/13 HIV-uninfected and 1/12 HIV-TB patients responded. Interestingly, by cytometry we showed that CD4^+^ T-cells response to HBHA was significantly impaired in the HIV-infected subjects with TB or LTBI compared to the HIV-uninfected subjects. The phenotype of HBHA-specific CD4 T-cells showed a predominantly central memory (CM) and effector memory (EM) phenotype without differences among the groups. Differently, HBHA-specific CD8^+^ T-cells, showed mainly a CM and naïve phenotype in LTBI group while TB, HIV-LTBI and HIV-TB groups were characterized by EM or terminally differentiated phenotypes. Interestingly, differently than what observed for RD1, the cytokine profile of HBHA-specific T-cells evaluated by cytometry showed that the CD4^+^ T-cells were mostly monofunctional. Conversely, CD8-specific T-cells were mostly monofunctional for both HBHA and RD1 stimulations.

**Conclusions:**

These results characterize the impact of HIV infection in CD4- and CD8-specific response to HBHA in both LTBI and TB patients. HIV infection impairs the CD4 response to HBHA and likely this may lead to an impairment of TB control.

## Introduction

Among the infectious diseases affecting humankind, human immunodeficiency virus (HIV) infection and tuberculosis (TB) rank firsts in terms of mortality and morbidity. Of the 10.4 million new TB cases estimated in 2015, 1.2 million were among people living with HIV and to the appraised 1.4 million TB deaths in the same year, an additional 0.4 million deaths resulting from TB disease among HIV subjects were reckoned [1]. Serious concern is also raising the increase in HIV incidence in TB endemic countries in South-East Asia and Russia [2;3].

HIV is known to impair host immune functions involved in controlling *Mycobacterium tuberculosis* (*Mtb)* infection in a number of ways [4]. Within the granulomas, HIV has been shown to promote cellular dysfunctions on CD4^+^ and CD8^+^ T-cells and macrophages, causing bacterial dissemination and TB reactivation [5]. Recent studies on humanized mice infected with HIV provided *in vivo* experimental evidences that pro-inflammatory responses in pulmonary TB are associated with poorly formed granulomas and increased disease severity [6]. TB disease in HIV-infected patients is characterized by reduced cavitation, increased bacillary dissemination and load, higher risk of developing drug resistance and poorer outcomes [7]. It is also known that immune impairment resulting from HIV-infection is responsible for the increased risk of *de novo Mtb* infection [8] and that HIV may affect the immunological diagnosis of *Mtb* infection [9–11]. Understanding how HIV infection shapes host immune responses against specific *Mtb* antigens at systemic level may pave the way for the development of improved immunodiagnostic tools with value for TB disease [12–15].

Immunological diagnosis of *Mtb* infection may be useful to identify subjects at risk of developing TB disease, particularly in high risk groups such as HIV-infected subjects [12]. RD1-based Interferon-(IFN)γ Release Assays (IGRAs) have been widely used in the last two decades and have demonstrated improved performance over the classical Mantoux test, although these assays cannot distinguish LTBI from active TB [9]. Among the *Mtb* antigens used in blood tests, the heparin-binding haemagglutinin (HBHA) emerged as a promising candidate with prognostic potential, since a negative response in HBHA-based IGRAs, among *Mtb*-infected subjects, has been shown by several groups to correlate with active TB disease rather than LTBI [16–21]. Here we aim to investigate the impact of HIV infection in CD4- and CD8-specific response to HBHA in both LTBI and TB patients, as a tool to evaluate the characteristics of the cells associated with a loss of *Mtb* containment.

## Materials and methods

### Study population and sample collection

**Fig 1:**
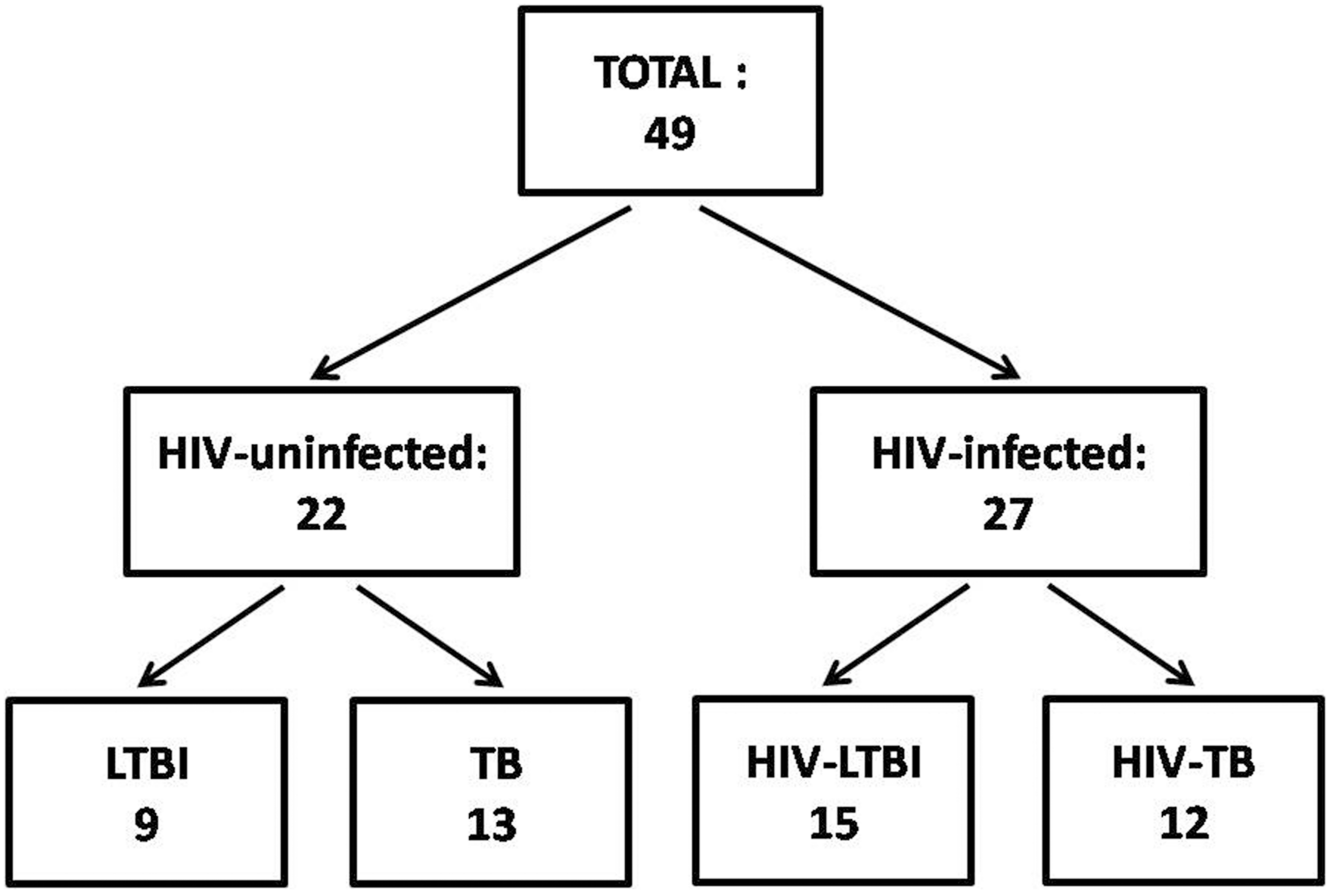
Flow chart of the patients enrolled for the study. Forty-nine *Mtb*-infected subjects were prospectively enrolled for the study. Twenty-two patients out of the total were HIV-uninfected, for 13 of these a diagnosis of active TB was made and 9 resulted LTBI. Among the remaining 27 HIV-infected patients, all naïve to ART, 12 were diagnosed as HIV-TB and 15 resulted HIV-LTBI. Footnotes: HIV: human immunodeficiency virus; TB: tuberculosis; LTBI: latent TB infection.

We conducted the study at the National Institute for Infectious Diseases (INMI) L. Spallanzani which was approved by the INMI Ethical Committee (approval number 34/2011). The participation to the study required the signature of a consent. The patients were prospectively enrolled (from 2012 to 2015). HIV-infected naïve to ART patients were diagnosed for HIV infection for the first time at enrollment but the infection was acquired prior 6 months. Active TB (HIV-TB and TB) patients were enrolled within the first week of TB-specific treatment. Active TB microbiologically diagnosed was defined based on the *Mtb* isolation from sputum culture. Microbiological TB was characterized by first line *Mtb* drug-sensitive isolates. LTBI patients were defined by QFT-IT positivity (Qiagen, Hilden, Germany) and absence of clinical symptoms, microbiological and chest X-ray evidence of lesions indicative of active TB. Preventive TB treatment was offered to all LTBI patients at the time of the study recruitment. Among the 49 patients enrolled, 22 (45%) were HIV-uninfected (13 active TB and 9 LTBI) and 27 (55%) were HIV-infected (12 HIV-TB and 15 HIV-LTBI) (Fig 1). The majority of the HIV-infected patients correspond to those analyzed in the our previous work. In particular, all among HIV-TB patients and 14/15 among the HIV-LTBI subjects [21]. Demographic and clinical characteristics of the subjects enrolled in the study are shown in Table 1. Around 75% of the enrolled cohort were BCG-vaccinated; regarding the origin, almost 36% were from Eastern Europe, 24% from Italy and 22% from South America. No significant difference in age gender and QFT-IT results was observed comparing all groups (Table 1); in contrast, the CD4^+^ and CD8^+^ T-cell counts and HIV-RNA copy numbers significantly differed among the HIV-infected groups analyzed (Table 1).

**Table 1:** Demographic and clinical characteristics of the subjects enrolled in the study.

	HIV-uninfected		HIV-infected			
	TB	LTBI	HIV-TB	HIV-LTBI	Total	*p* value
N (%)	13 (26.5)	9 (18.4)	12 (24.5)	15 (30.6)	49 (100)	
Median Age (IQR)	30.0 (25.0–46.0)	48.0 (34.5–60.0)	40.0 (30.3–44.0)	38.0 (35.0–40.0)	38.0 (30.5–44.5)	0.3^a^
Female gender (%)	1 (7.7)	1 (11.1)	0	3 (20.0)	5 (10.2)	0.4^b^
Origin (%)						0.001^b^
Italy	2 (15.4)	6 (66.7)	3 (25.0)	1 (6.7)	12 (24.5)	
Eastern Europe	9 (69.2)	1 (11.1)	5 (41.6)	3 (20.0)	18 (36.7)	
Asia	1 (7.7)	-	-	-	1 (2.0)	
Africa	1 (7.7)	2 (22.2)	2 (16.7)	2 (13.3)	7 (14.3)	
South America	-	-	2 (16.7)	9 (60.0)	11 (22.5)	
BCG status (%)						0.008^b^
Vaccinated	11 (84.6)	3 (33.3)	9 (75.0)	14 (93.3)	37 (75.5)	
Unvaccinated	2 (15.4)	6 (66.7)	3 (25.0)	1 (6.7)	12 (24.5)	
QFT-IT (%)						0.24^b^
Positive	11 (84.6)	9 (100)	10 (83.4)	15 (100)	45 (91.8)	
Negative	2 (15.4)	-	2 (16.7)	-	4 (8.2)	
CD4^+^ T-cell/mm^3^ # Median (IQR)	-	-	128.5 (41.0–266.5)	666 (341.0–1075.0)	329 (110.0–767.0)	0.0001^c^
CD8^+^ T-cell/mm^3^ # Median (IQR)	-	-	532.5 (153.3–815.0)	1053 (884.0–1352.0)	877 (481.0–1287.0)	0.0001^c^
HIV-RNA (cp/μl) # Median (IQR)	-	-	60542.0 (7451.8–610654.3)	6878.0 (1427.5–24809.0)	16689.0 (1884–150192.0)	0.035^c^

HIV: human immunodeficiency virus; LTBI: latent tuberculosis infection; TB: active tuberculosis; IQR: interquartile range; BCG: Bacillus Calmette et Guérin; QFT-IT: QuantiFERON-TB Gold (QFT^®^) in tube;

^#^: only available for HIV-infected patients.

^a^ Kruskal-Wallis test.

^b^ Chi-square test.

^c^ Mann-Whitney.

### Stimuli for in vitro tests

We stimulated the peripheral blood mononuclear cells (PBMC) for 16 hours with *Mtb*-specific antigens: a) RD1 proteins that consist in a mix of recombinant proteins ESAT-6 and CFP-10 (Lionex, Braunschweig, Germany) used at 4 μg/ml (LPS contamination was for ESAT-6 less than 0.05 IU/mg and for CFP-10 was equal to 66.7 IU/mg, as reported by the manufacturer); b) a recombinant methylated HBHA which was purified from *M*. *smegmatis* pMV3-38 as previously described [22] (LPS contamination was 0.60 IU/mg of protein) and used at a final concentration of 5 μg/ml [19]. The unrelated antigen CMV lysate (strain AD169) (Experteam, Venice, Italy) was used at 5 μg/ml while as positive control the SEB (Sigma, St Louis, MO, USA) was used at 200 ng/ml.

A costimulation with anti-CD28 and anti-CD49d monoclonal antibodies (mAb) at 2 μg/ml each (BD Bioscence, San Jose, USA) was performed. The fluorescently conjugated mAb used were those listed below: anti-CD3 allophycocyanin (APC)-Vio770, anti-CD8 VioBlue, anti-CD4 peridinin chlorophyllprotein (PerCP)-Vio700, anti-CD45RA phycoerythrin (PE)-Vio770, anti-CCR7 VioGreen, anti-IFN-γ APC, anti-TNF-α fluorescein isothiocyanate (FITC) and anti-IL-2 PE (all mAb from Miltenyi Biotec).

### QuantiFERON-TB Gold In-tube (QFT-IT)

We used the commercially available IGRA QuantiFERON-TB Gold In-tube (QFT-IT) (Qiagen) as indicated by the manufacturer’s instructions using TB antigen, Mitogen (as positive control) and Nil (as negative control). The TB antigen tube contains the overlapping peptides from CFP-10 and ESAT-6 and TB7.7. The cut-off value for a positive test was ≥0.35 IU/ml, while an indeterminate score was assigned if the IFN-γ response to the Mitogen after subtracting the Nil was <0.5 IU/ml or if the Nil was >8 IU/ml. The data are presented as IU/ml after subtraction of the Nil.

### HBHA-IGRA

Whole blood (0.250 ml heparinised whole blood per well) was seeded in a 48-well plate (Corning Costar, Corning Incorporated, New York, NY, USA) for 16–20 hours and left unstimulated or treated with the recombinant methylated HBHA which was purified from *M*. *smegmatis* pMV3-38 [22] (LPS contamination was 0.60 IU/mg of protein) and used at a final concentration of 5 μg/ml. The IFN-γ production was measured on the harvested plasma by the commercial ELISA used within the QFT-IT. Data are presented as IU/ml after subtraction of the unstimulated condition.

### Intracellular staining assay (ICS)

We isolated fresh PBMC using Ficoll density gradient centrifugation, and 1x10^6^ cells/ml were cultured overnight with the single stimuli and the following costimuli as anti-CD28 and anti-CD49d monoclonal antibodies at 2 μg/ml each (BD Bioscence) at 37°C and 5% CO_2_ in 10% fetal bovine serum (PAA Laboratories GmbH, Pasching, Austria) in RPMI-1640 (Gibco, CA, USA). To prevent cytokine secretion the BD GolgiPlug was added after 1 hr of stimulation. After 16 hrs of incubation the ICS was performed. Unstimulated PBMC provided as a negative control. PBMC were first stained with mAb for surface markers, fixed in 4% paraformaldehyde and permeabilized with PBS-1% BSA -0.5% saponin -0.1% NaN_3_ and then stained with mAb for intracellular cytokines. Cells were fixed again in 2% paraformaldehyde, and at least 200,000 lymphocytes were acquired using a FACSCanto II flow cytometer (BD Biosciences).

### Flow cytometry data analysis

The FlowJo (Tree Star Inc., San Carlos, CA), Pestle and SPICE softwares (provided by Dr. Roederer, Vaccine Research Center, NIAID, NIH, USA, 28), were used to analyze the multiple-parameter flow cytometry data. According to forward and side scatter plots, the cells were gated and, as previously described [23], the frequency of single, double and triple cytokines expressed by CD4^+^ and CD8^+^ T-cells was evaluated using Boolean combination gates. Background cytokine production in the negative control of the ICS assay was subtracted from each stimulated condition. A positive cytokine response was defined as at least twice the background. A frequency of any cytokine-producing T-cells (IFNγ and/or TNFα and/or IL2) of at least 0.03% was considered as a positive CD4^+^ and CD8^+^ T-cell response corresponding to at least 30 analyzed events.

In preliminary experiments, we tested the quality of our fluorescent antibody using isotype control. Moreover, to properly interpret flow cytometry data, we performed the Fluorescence Minus One (FMO) Control. FMO control contains all the fluorochromes in a panel, except for the one that is being measured. Using FMO it is possible to identify any potential spread of the fluorochromes into the channel of interest.

According to the expression of the surface markers CD45RA and CCR7, the phenotypical analysis of different antigen-memory response of CD4^+^ and CD8^+^ T-cells was evaluated by flow cytometry. The memory status of antigen-specific CD4^+^ or CD8^+^ T-cells was evaluated on differently gated CD4^+^ and CD8^+^ T-cells respectively. In particular, the phenotypical analysis was performed within the gates defined as total CD4 T-cell response and total CD8^+^ T-cell response, identifying CD4^+^ and CD8^+^ T-cells only in the subjects with a positive cytokine response to the antigens.

The analysis was performed the same day of enrolment for every patients. The frequency of cytokine expression (IFNγ, TNFα and IL2) and the phenotype was evaluated by flow cytometry in the Ag-specific CD4^+^ and CD8^+^ T-cells.

Independent blinded analysis was then performed using the same gating strategy by two authors (TC and after, by EP). Concordance of the analyses was 90% and agreement was achieved by discussion.

### Statistical analysis

As previously described [23], the data were analyzed using SPSS software (Version 19 for Windows, Italy SRL, Bologna, Italy). The medians and interquartile range (IQR) were calculated for continuous measures, the Mann-Whitney or Kruskal-Wallis tests were used to analyze unpaired data. The Chi-square test was used for non-continuous measures. P values as ≤0.05 were considered significant.

## Results

### HBHA-IGRA

**Fig 2:**
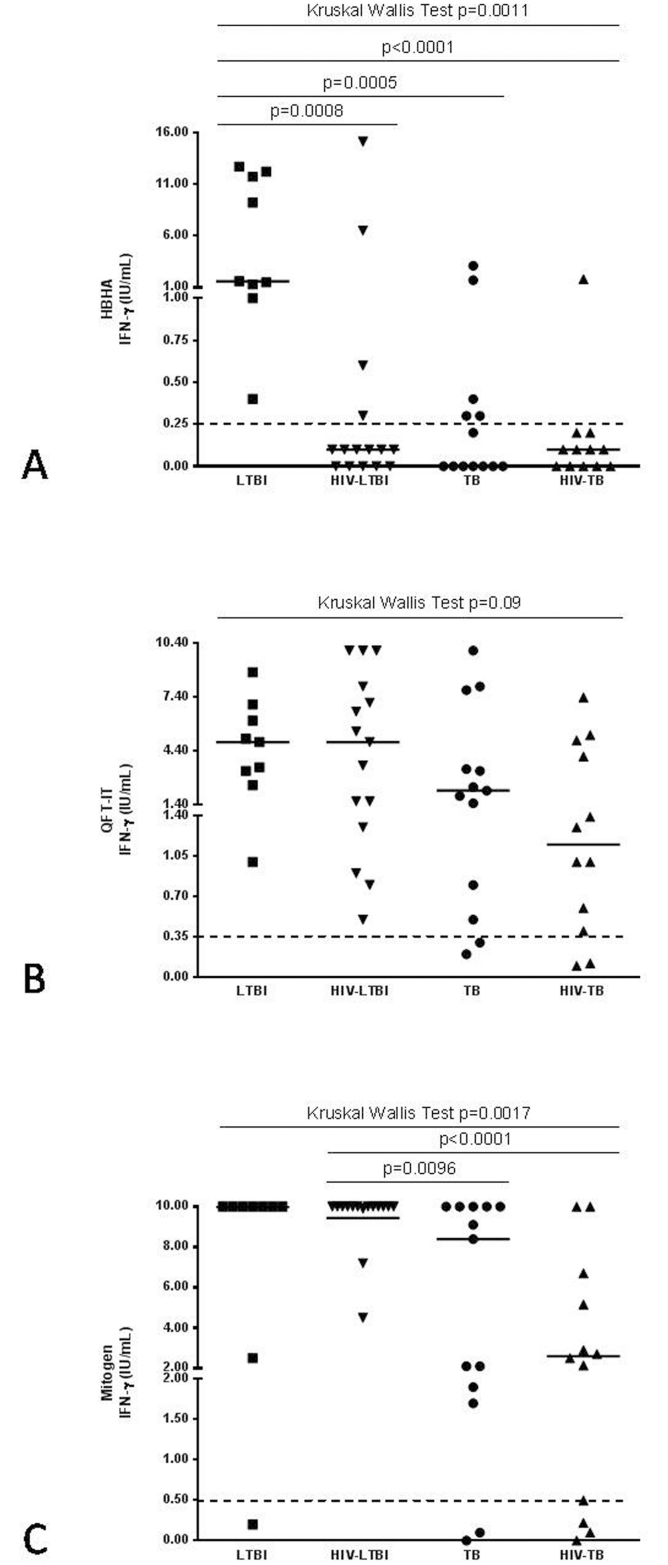
IFNγ-response to different *Mtb*-antigens in patients enrolled for the study. IFNγ-response to different *Mtb*-antigens was evaluated by short-term stimulation of whole blood in *Mtb*-infected patients with or without HIV infection. IFNγ from day-1 supernatants were evaluated by commercial ELISA in response to HBHA (A), QFT-IT antigen (B) and Mitogen (C). The square represents LTBI group, the triangle with the bottom tip represents HIV-LTBI group, the dot represents active TB group and the triangle with the top tip represents HIV-TB group. The data are shown after the subtraction of the results obtained in the unstimulated samples. Horizontal lines indicate the median of IFNγ production. Dotted lines indicate the cut-off for either QFT-IT or Mitogen as indicated by the suppliers and for HBHA antigen as for previously shown results [24;25]. Footnotes: HIV: human immunodeficiency virus; TB: tuberculosis; LTBI: latent TB infection; HBHA: heparin-binding haemagglutinin; QFT-IT: QuantiFERON-TB Gold (QFT^®^) in tube; IFN: interferon; IU: International Unit.

Regarding the response to HBHA stimulation, among the LTBI individuals, which by definition were QFT-IT positive (Fig 2B), all 9 HIV-uninfected LTBI subjects, scored positive, while among the 15 HIV-LTBI patients only 4 responded. Within the 13 HIV-uninfected active TB patients, only 5 scored positive whereas among the 12 HIV-TB patients, only one responded *in vitro* to HBHA (Fig 2A). IFNγ release to QFT-IT was not significantly different between the four matched groups, confirming that QFT-IT cannot distinguish LTBI from active TB in patients with or without HIV-co-infection (Fig 2B). IFNγ release following Mitogen stimulation was significantly lower in TB and HIV-TB groups compared to HIV-LTBI, suggesting that TB status induced a weaker overall T-cell response (Fig 2C).

### Polyfunctional or monofunctional response to HBHA

**Fig 3:**
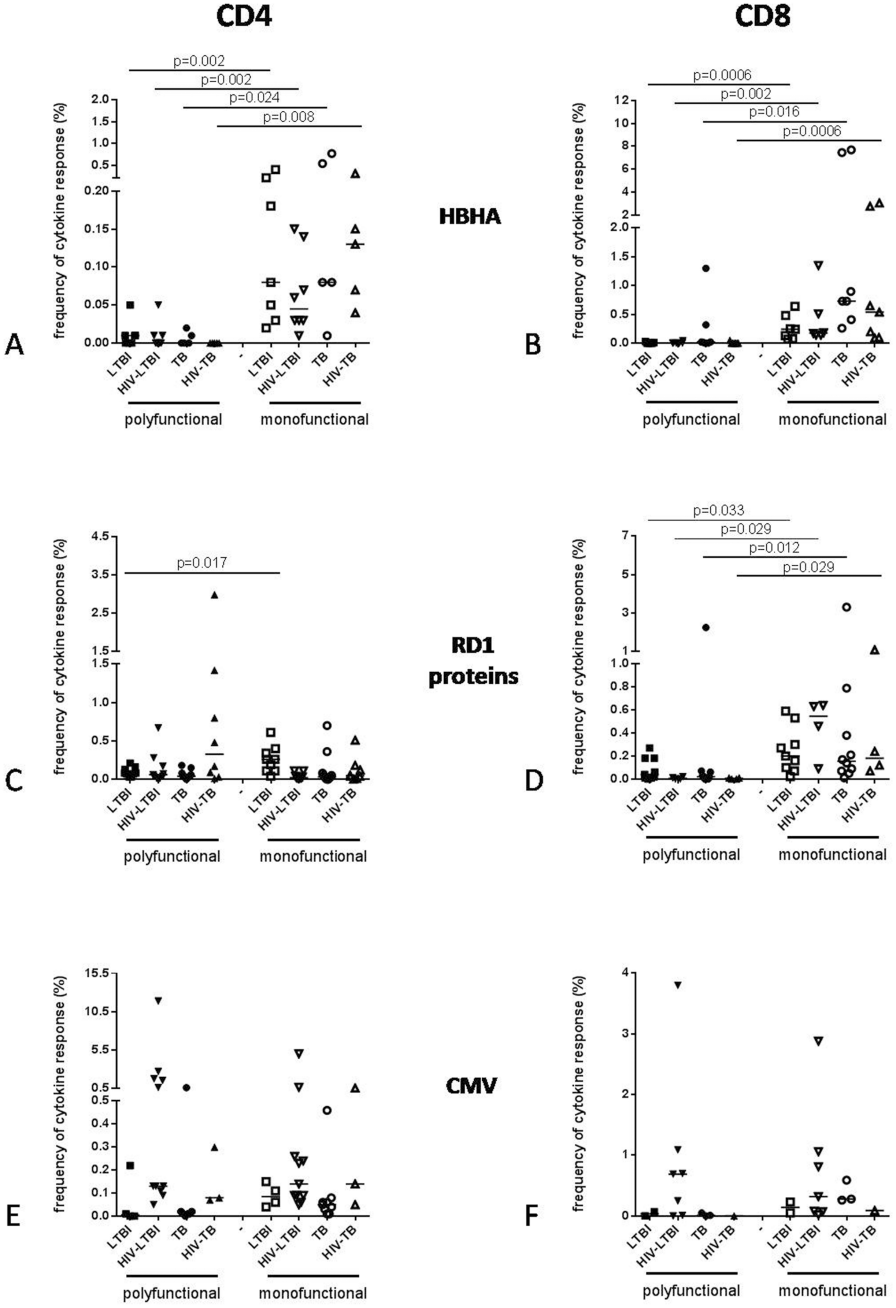
Analysis of polyfunctional and monofunctional response to different antigens of CD4^+^ and CD8^+^ T-cell subsets in patients enrolled for the study. The graphs show the frequency of polyfunctional (more than one cytokine) and monofunctional (one cytokine) response, evaluated by flow cytometry, to HBHA (A, B), RD1 proteins (C, D) and CMV (E, F) of CD4^+^ and CD8^+^ T-cell subsets in the *Mtb*-infected patients with or without HIV infection. A positive cytokine response was defined as at least twice the background. A frequency of any cytokine-producing T-cells (IFNγ and/or TNFα and/or IL2) of at least 0.03% was considered as a positive CD4 and CD8 T-cell response. The horizontal lines represent the median; filled symbols represent polyfunctional T-cells, open symbols represent monofunctional T-cells. Statistical analysis was performed using the Mann-Whitney test and p value was considered significant if < 0.05. Footnotes: HIV: human immunodeficiency virus; TB: tuberculosis; LTBI: latent TB infection; HBHA: heparin-binding haemagglutinin; RD: region of difference; CMV: cytomegalovirus.

To characterize the T-cell response to HBHA, polyfunctional (more than one cytokine) and monofunctional (one cytokine) responses to HBHA antigen were analyzed in CD4^+^ and CD8^+^ T-cell subsets (Fig 3). RD1 proteins and CMV antigens were included as controls. Considering the CD4 T-cell response, all groups showed a higher frequency of monofunctional T-cells than the polyfunctional, in response to HBHA antigen (LTBI p = 0.002; HIV-LTBI p = 0.002; TB p = 0.024 and HIV-TB p = 0.008 respectively) (Fig 3A). Conversely, the CD4 T-cell responses to the other RD1-specific or recall antigens were found preferentially polyfunctional than monofunctional or without any statistical difference with the exception of the response to the RD1 proteins which was mainly monofuntional in LTBI (p = 0.017) (Fig 3C and 3E). The HBHA-specific CD8^+^ T-cell responses showed a higher frequency of monofunctional than polyfunctional T-cells in all groups (LTBI p = 0.0006; HIV-LTBI p = 0.002; TB p = 0.016 and HIV-TB p = 0.0006 respectively) (Fig 3B). Similar results were observed for the RD1-specific response but not for the CMV (Fig 3D and 3F).

Hence, while the RD1-specific responses may be polyfunctional, the HBHA-specific CD4 T-cells are selectively monofunctional. Conversely, CD8-specific T-cells are mostly monofunctional for both HBHA and RD1 stimulations.

### Cytokine profile of CD4^+^ and CD8^+^ T-cells in response to HBHA antigen

**Fig 4:**
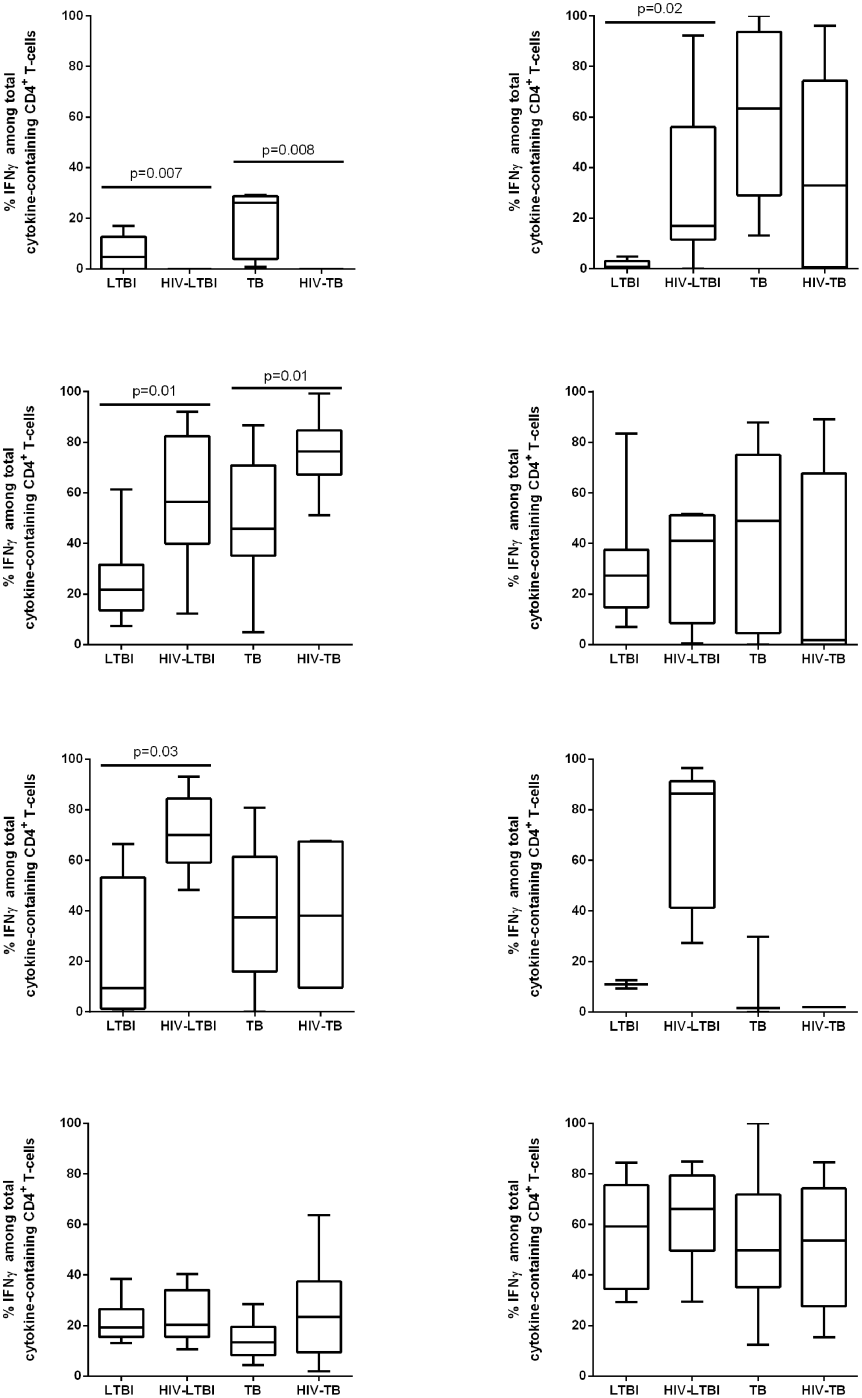
IFNγ profile of CD4^+^ and CD8^+^ T-cells in response to different stimuli. The IFNγ cytokine profile of CD4^+^ and CD8^+^ T-cells was analyzed by ICS. In particular, we evaluated the “total IFNγ response” and then calculated the proportion of this response within the total cytokine response (IFNγ and/or TNFα and/or IL2). PBMC were stimulated overnight with HBHA (A, B), RD1 proteins (C, D), CMV (E, F) and SEB (G, H) and analyzed within the CD4^+^ and CD8^+^ T-cells by flow cytometry. A-H: The bar graphs show the proportion of IFNγ-expressing cells within the CD4^+^ and CD8^+^ T-cells over the total responding T-cells in the different groups analyzed. The horizontal lines represent the median. Statistical analysis was performed using Mann-Whitney test and p value was considered significant if < 0.05. A, C, E, G) Proportion of total IFNγ-producing CD4^+^ T-cells in response to each stimulus; B, D, F, H) Proportion of total IFNγ-producing CD8^+^ T-cells in response to each stimulus. Footnotes: HIV: human immunodeficiency virus; TB: tuberculosis; LTBI: latent TB infection; HBHA: heparin-binding haemagglutinin; RD: region of difference; CMV: cytomegalovirus; SEB: staphylococcal enterotoxin B; IFN: interferon.

The cytokine profiles of CD4^+^ and CD8^+^ T-cells were analyzed by evaluating the proportion of each cytokine (IFNγ and/or TNFα and/or IL2) within the total antigen response using the Boolean gate combinations (S1 Fig). To better understand the cell source of IFNγ production in the groups studied, we evaluated the “total IFNγ-response” and used this value to calculate its proportion over the “total cytokine response” (Fig 4). HIV significantly impaired the response to HBHA in CD4^+^ T-cells both in subjects with LTBI (p = 0.007) and in those with TB disease (p = 0.008).

Moreover, among the LTBI group, IFNγ was expressed mainly by CD4 T-cells, whereas in TB, it was expressed both by CD4^+^ and CD8^+^ T-cells (Fig 4A and 4B). Moreover, in LTBI subjects, we found that, even if they were globally more monofunctional than polyfunctional responses, CD4 T-cells expressing IFNγ were either mono-, bi- or tri-functional, whereas in TB the CD4 T-cells expressing IFNγ were mono- or bi-functional (S1 Fig).

Interestingly, in HIV-infected subjects, the IFNγ-induced HBHA response was mediated only by CD8^+^ T-cells (Fig 4B), that were mainly monofunctional (S1 Fig).

Taken together these cytometry results indicate that HIV has a negative impact on the ability of CD4 to respond to HBHA.

### Cytokine profile of CD4^+^ and CD8^+^ T-cells in response to different antigens

To assess the specificity and quality of the T-cell responses directed against HBHA, we analyzed the CD4^+^ and CD8^+^ T-cell-responses elicited by other *Mtb*-specific antigens as RD1 proteins, an unrelated antigen as CMV and mitogenic stimulus as SEB (Fig 4C–4H).

Previously, we have described the comparisons between TB and LTBI with or without HIV infection [26;27], therefore in this study we focused to analyze the relation between LTBI vs HIV-LTBI and TB vs HIV-TB groups.

Regarding the other stimuli, IFNγ was expressed by CD4^+^ T-cells. In particular a significant higher proportion of IFNγ expressing cells in response to RD1 proteins was associated with HIV (in respect to LTBI, or TB, p = 0.01 for both comparisons). Regarding the response to CMV, this was significantly associated with HIV-LTBI compared to LTBI (p = 0.03). Within the CD8^+^ T-cells, no significant differences were observed among the groups in response to RD1 and SEB. To note that the CMV responses were mediated by CD8^+^ T-cells only in HIV-LTBI (Fig 4F). The majority of the CD8-mediated responses were monofunctional (S1 Fig).

Taken together these cytometry results indicate that HIV has a selective negative impact on the ability of CD4^+^ T-cells to respond to HBHA, not to the other stimuli studied.

### Memory status of HBHA antigen-response of CD4^+^ and CD8^+^ T-cells

**Fig 5:**
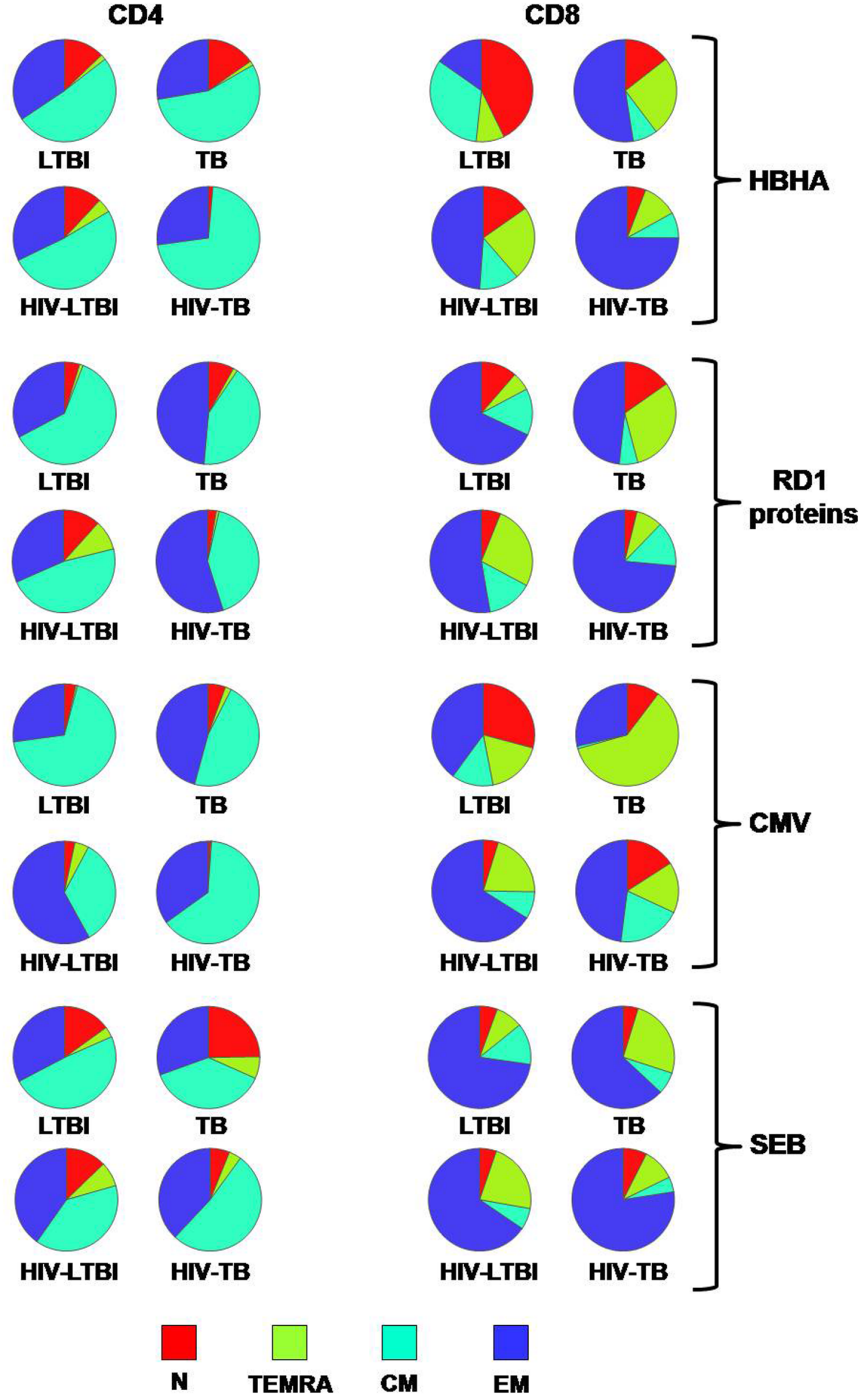
Memory status of CD4^+^ and CD8^+^ T-cells in response to different stimuli. Memory status of CD4^+^ and CD8^+^ T-cell response was evaluated by flow cytometry according to the surface expression of CD45RA and CCR7 in the gate of the total CD4^+^ and CD8+ T-cell response. We defined naïve (N) as CD45RA^+^ CCR7^+^, terminally differentiated effector memory T-cells (T_EMRA_) as CD45RA^+^ CCR7^-^, central memory (CM) as CD45RA^-^ CCR7^+^ and effector memory (EM) as CD45RA^-^ CCR7^-^. The pie charts represent the proportion of N, T_EMRA_, CM and EM CD4^+^ and CD8^+^ T-cells in the different groups analyzed in response to overnight stimulation with the different stimuli. For statistical comparison and detailed evaluation, please see S2 Fig.

To further dissect the T-cell populations specific for HBHA, a characterization of their immune phenotype was carried out [27]. CD4^+^ and CD8^+^ T-cells were analyzed depending on their memory status as naïve (N), terminally differentiated (T_EMRA_), central memory (CM) and effector memory (EM) (Fig 5 and S2 Fig). Within the CD4 T-cell-response, we did not find any memory status associated with *Mtb*-infected and HIV coinfected status. The phenotype of HBHA-specific T-cells showed a predominantly central memory (CM) and effector memory (EM) phenotype in CD4 T-cells, as previously reported [28] without differences among the groups (Fig 5). Conversely, among the CD8^+^ T-cells a significantly higher proportion of T_EMRA_ were found in the TB group compared to the HIV-TB group (p = 0.048, S2 Fig). Moreover, increased proportion of EM and T_EMRA_ CD8^+^ specific cells were found in the HIV-LTBI compared to the LTBI (both p = 0.042) (Fig 5 and S2 Fig).

Taken together these results indicate the absence of main differences in the memory status of HBHA-specific CD4^+^ T-cells among the group studied; conversely, concerning the HBHA-specific CD8^+^ T-cells, LTBI show mainly a N or CM phenotype, while the TB, HIV-LTBI and HIV-TB groups are characterized by EM or T_EMRA_ phenotypes.

### Memory status of different antigen-response of CD4^+^ and CD8^+^ T-cells

To better define the specificity of the memory status results obtained with HBHA antigens, a comparison with those elicited by RD1-specific responses, unrelated antigens (CMV) and mitogenic stimulus (SEB) was carried out (Fig 5 and S2 Fig). Comparing the patients with active TB, within the CD4^+^ T-cell-response to RD1 proteins and SEB, a N status was associated with TB group (p = 0.032; p = 0.026 respectively) (Fig 5 and S2 Fig). Interestingly in TB we observed a trend of a diminution of CM with a rise of EM as previously shown (Fig 5 and S2 Fig) [26].

Within the LTBI, regarding the response to CMV, although the proportion is very low, a T_EMRA_ phenotype and an EM status associated with HIV-LTBI patients (p = 0.005; p = 0.021 respectively); while, a CM status was associated with the LTBI group (p = 0.009) (Fig 5 and S2 Fig).

Regarding CD8^+^ T-cell-response to SEB among the patients with active TB, a T_EMRA_ phenotype was associated with TB group (p = 0.009) (Fig 5 and S2 Fig). Within the LTBI groups, analyzing the CD8^+^ T-cell-response to RD1 proteins and SEB, we found a T_EMRA_ status associated with HIV-LTBI patients (p = 0.031; p = 0.009 respectively) (Fig 5 and S2 Fig).

## Discussion

In previous studies, we and others have shown that a positive response to HBHA-based IGRAs in immunocompetent subjects correlates mainly with Mtb containment and LTBI, suggesting that HBHA may serve as an immunological correlate of protection [16;17;19;28; 29]. The potential usefulness of a such HBHA-based blood test would be of the greatest relevance in Mtb-infected patients at higher risk of developing active disease, as the HIV-infected subjects. Indeed, we recently confirmed that a positive HBHA-based IGRA in HIV-LTBI correlates with Mtb containment [21]. In this study, we aimed to characterize the immune phenotypes of the HBHA-specific CD4^+^ and CD8^+^ T-cell responses in Mtb-infected patients, with or without HIV infection. Our findings indicate that in those with LTBI, IFNγ in response to HBHA is expressed mainly by CD4^+^ T-cells, whereas in those with TB, by both, the CD4^+^ and CD8^+^ T-cells. The HBHA-specific CD4^+^ T-cells in the LTBI were found to be either monofunctional (IFNγ+), double functional (IFNγ+ TNFα+) or triple functional (IFNγ+ IL2+ TNFα+), as previously shown in the HIV-uninfected subjects [30]. CD4 T-cells expressing TNFα were not significantly different among the groups, as previously shown [30]. The phenotype of HBHA-specific CD4 T-cells showed a predominantly CM and EM phenotype without differences among the groups. Regarding the CD8^+^ T-cells, the HBHA-specific cells were mainly monofunctional (IFNγ+ or TNFα+). TNFα-expressing CD8^+^ T-cells were not significantly different among the groups, as previously shown [30]. Regarding the phenotype, CD8^+^ T-cells were characterized either by a terminally differentiated (TEMRA) or effector memory (EM) phenotype in TB, HIV-LTBI and HIV-TB but not in the LTBI. Moreover, interestingly, in the HIV-infected subjects, the HBHA-specific IFNγ-response was selectively mediated by CD8^+^ T-cells, although the CD4^+^ T-cells did respond to all the other stimuli tested, as RD1 proteins, CMV and SEB. This is likely due to the baseline low frequency of the HBHA-specific responses within the CD4^+^ T cells compared to other *Mtb* antigens as RD1 which lead to its dramatic loss after HIV infection. Altogether, these results provide for the first time a concomitant fine characterization of the HBHA-specific immune response in both CD4^+^ and CD8^+^ T-cells in HIV-infected and uninfected patients.

Seminal studies and more recent evidences suggest that polyfunctional CD4^+^ and CD8^+^ T-cells secreting IFNγ in combination with other cytokines, such as TNFα and IL2, may be key players in the protective immunity against TB [26;31–35]. For these reasons, *Mtb* antigen-specific polyfunctional T-cells have been studied deeply either in observational studies involving patients at different TB stages or in vaccine-based studies [32;35;36]. However, conflicting results have been generated. Therefore it remains to be determined whether polyfunctional T-cells represent a marker of protective immunity or of disease activity 27; 32–36. In fact, polyfunctional CD4 T-cells in response to RD1 antigens were found at similar or higher levels in HIV-TB compared to HIV-LTBI, though in the HIV-TB group their ability to express more than one cytokine decreased with the increase in HIV-1 viral load [27;37;38]. In this study, we observed that, HBHA-specific triple functional CD4^+^ T-cells associated with LTBI, as previously shown [30]. Moreover, among the CD8^+^ T-cells, we did not find IFNγ monofunctional CD8^+^ T-cells in LTBI, differently from what observed in the other groups. Since the LTBI group showed higher level of IFNγ in the IGRA-based on HBHA, it follows that the CD4 T-cells positive for IFNγ are likely those mainly responsible for the higher levels of IFNγ production observed by ELISA in this group. However, it must be noted that previous studies failed to find a direct correlation between ICS and ELISA, and therefore it remains to be determined how much IFNγ is secreted by the IFNγ+ CD4^+^ and CD8^+^ T-cells specific for HBHA [39]. Nevertheless, these findings provide additional support to the potential protective role of HBHA-specific CD4^+^ T-cells against *Mtb* and highlight their importance in HIV infection where a specific loss is observed. These results are further supported by experiments done in animal models [17;40;41].

Interestingly, we found that HIV infection significantly impairs the IFNγ expression in response to HBHA in the CD4^+^ T-cells. This is remarkable because the same CD4^+^ T-cells do respond to different *Mtb* antigens as RD1 or recall antigens as CMV or to a promiscuous antigen as SEB. Another report described the response to HBHA in HIV-infected subjects [28;42]. However the experimental system used is different compared to that used in the present study because it involved PBMC instead of whole blood, it included the *in vitro* stimulation with IL-7 and the use of a different HBHA (from *M*. *bovis*). Moreover, a small cohort of only 6 HIV-TB patients was evaluated and the status of LTBI was not defined by a positive response to QFT-IT, as we did here. Nevertheless, in both studies, a proportion of HIV-TB responding to the HBHA-IGRA was described. Regarding the different results obtained from the literature, it is important to note that here we found lower cytokines frequencies in response to HBHA compared to others [30], likely due to the shorter time of stimulation we used (1 day) in respect to that reported by others (5 days) beside all the experimental differences reported above. This experimental specificities may explain the lack of response to HBHA in the CD4^+^ T cells from HIV-infected subjects.

CD8^+^ T-cells are known to play a role during *Mtb* infection by secreting cytokines and molecules with cytotoxic activity [43] and anti-mycobacterial activity [44]. An inverse correlation between TB disease therapy success and *Mtb*-specific CD8^+^ T-cells capable to secrete IFNγ has been observed [32] indicating that the RD1-specific-CD8^+^ cells correlate with *Mtb* load [27;45]. Hence, similarly to what observed for RD1 antigens, an increase in peripheral IFNγ-secreting CD8^+^ T-cells specific for HBHA inversely correlates with the ability to contain *Mtb in vivo*. Previous findings reported that HBHA-specific CD8^+^ T-cells are involved in the containment of *Mtb* infection in LTBI, though the ability to restrict mycobacterial growth was associated with CD8^+^ T-cells secreting perforin [23]. Interestingly, the HBHA-specific, perforin-producing CD8 T lymphocytes were distinct from the IFNγ-producing CD8^+^ T-cells [23], suggesting that the latter population of CD8^+^ T-cells may not be involved in the control of *Mtb* infection as previously suggested [43].

Among the CD4^+^ T-cells responding to HBHA, the response was mainly mediated by CM and EM, as previously reported [28] without differences among the groups. Differently, among the CD8^+^ T-cells an EM and a T_EMRA_-status were associated with TB and a N phenotype was associated with LTBI. Moreover, a significantly higher proportion of CD8^+^ T-cells showing a T_EMRA_-phenotype was found in the TB group compared to the HIV-TB group, and in the HIV-LTBI compared to the LTBI. The different phenotype likely reflects the different *Mtb* load present in the groups studied.

A potential limitation of the present study is the relatively small number of the subjects analyzed. However, to our knowledge, this is the first study in which it has been reported the concomitant characterization of the immune HBHA-specific response among HIV-infected and uninfected subjects with active TB or LTBI. Moreover, considering the literature, the number of subjects enrolled is comparable to those described in similar reports. In addition, the design of the study was very systematic in terms of: groups analyzed (HIV-TB vs TB and HIV-LTBI vs LTBI), experimental tools used (*Mtb* specific and unrelated recall antigens employed), integrity and reproducibility of the results obtained (cytometry data were analyzed by two independent laboratory operators), evaluation of the cytokine profile and memory status in both CD4^+^ and CD8^+^ T-cell subsets. Finally, LTBI was defined by QFT-IT positivity and absence of clinical and microbiological symptoms and chest X-ray evidence of lesions indicative of active TB, definition used in several reports including those written by the WHO [46]. This definition has been challenged by a very interesting approach using a stratification of patients based on the concomitant evaluation of the response to HBHA and ESAT-6 [47]. This approach, although fascinating, needs to be validated in larger cohorts of patients and in the meantime, we use the definition of LTBI widely employed [12;46;48–50].

In conclusion, these results significantly improve our knowledge of the human immune response to *Mtb* phase-dependent antigens in the long-term control of infection, on the impact of HIV infection in these responses and provide suggestions for designing vaccines based on *Mtb* latency antigen.

## Supporting information

S1 Fig Cytokine profile of CD4^+^ and CD8^+^ T-cells in response to different stimuli. PBMC were stimulated overnight with HBHA (A, B), RD1 proteins (C, D), CMV (E, F) and SEB (G, H) and analyzed by flow cytometry for intracellular production of IFNγ, TNFα and IL2. A-H: The bar graphs show the proportion of stimuli cytokine producing CD4^+^ and CD8^+^ T-cells over the total responding T-cells in the different groups analyzed. The horizontal lines represent the median; blue circles represent the LTBI group, red circles represent the HIV-LTBI group; green circles represent the TB group; orange circles represent the HIV-TB group. Statistical analysis was performed using Mann-Whitney test and p value was considered significant if < 0.05. A, C, E, G) Proportion of cytokine-producing CD4^+^ T-cells in response to each stimulus; B, D, F, H) Proportion of cytokine-producing CD8^+^ T-cells in response to each stimulus. Footnotes: HIV: human immunodeficiency virus; TB: tuberculosis; LTBI: latent TB infection; HBHA: heparin-binding haemagglutinin; RD: region of difference; CMV: cytomegalovirus; SEB: staphylococcal enterotoxin B; IFN: interferon; IL: interleukin; TNF: tumor necrosis factor.
(TIF)

S2 Fig Memory status of CD4^+^ and CD8^+^ T-cells in response to different stimuli. Memory status of CD4^+^ and CD8^+^ T-cell response was evaluated by flow cytometry according to the surface expression of CD45RA and CCR7 in the gate of total CD4 and CD8 T-cell response. We defined naïve (N) as CD45RA^+^ CCR7^+^, terminally differentiated effector memory T-cells (T_EMRA_) as CD45RA^+^ CCR7^-^, central memory (CM) as CD45RA^-^ CCR7^+^ and effector memory (EM) as CD45RA^-^ CCR7^-^. A-H: The bar graphs represent the proportion of N, T_EMRA_, CM, and EM CD4^+^ and CD8^+^ T-cells in the different groups analyzed in response to overnight stimulation with the different stimuli. The horizontal lines represent the median; blue circles represent the LTBI group, red circles represent the HIV-LTBI group; green circles represent the TB group; orange circles represent the HIV-TB group. Statistical analysis was performed using Mann-Whitney test and p value was considered significant if < 0.05. A, C, E, G) Phenotype of CD4^+^ T-cells in response to each stimulus; B, D, F, H) Phenotype of CD8 T-cells in response to each stimulus. Footnotes: HIV: human immunodeficiency virus; TB: tuberculosis; LTBI: latent TB infection; HBHA: heparin-binding haemagglutinin; RD: region of difference; CMV: cytomegalovirus; SEB: staphylococcal enterotoxin B; N: naïve; T_EMRA_: terminally-differentiated effector memory; CM: central memory; EM effector memory.
(TIF)

## References

[1] World Health Organization. Global Tuberculosis Report 2016. 2016.

[2] Pokrovskiy V. HIV epidemic in Russia and neighbouring countries. J.Int.AIDS Soc. 2014; 17:19502 doi: 10.7448/IAS.17.4.19502 25394011 10.7448/IAS.17.4.19502 PMC4224896

[3] Podlekareva DN, Efsen AM, Schultze A, Post FA, Skrahina AM, Panteleev A, et al Tuberculosis-related mortality in people living with HIV in Europe and Latin America: an international cohort study. Lancet HIV. 2016; 3:e120–e131. doi: 10.1016/S2352-3018(15)00252-0 26939735 10.1016/S2352-3018(15)00252-0

[4] Ronacher K, Joosten SA, Van CR, Dockrell HM, Walzl G, Ottenhoff TH. Acquired immunodeficiencies and tuberculosis: focus on HIV/AIDS and diabetes mellitus. Immunol.Rev. 2015; 264:121–37. doi: 10.1111/imr.12257 25703556 10.1111/imr.12257

[5] Diedrich CR, Flynn JL. HIV-1/mycobacterium tuberculosis coinfection immunology: how does HIV-1 exacerbate tuberculosis? Infect.Immun. 2011; 79:1407–17. doi: 10.1128/IAI.01126-10 21245275 10.1128/IAI.01126-10 PMC3067569

[6] Nusbaum RJ, Calderon VE, Huante MB, Sutjita P, Vijayakumar S, Lancaster KL, et al Pulmonary Tuberculosis in Humanized Mice Infected with HIV-1. Sci.Rep. 2016; 6:21522 doi: 10.1038/srep21522 26908312 10.1038/srep21522 PMC4808832

[7] Pollock KM, Montamat-Sicotte DJ, Grass L, Cooke GS, Kapembwa MS, Kon OM, et al PD-1 Expression and Cytokine Secretion Profiles of Mycobacterium tuberculosis-Specific CD4+ T-Cell Subsets; Potential Correlates of Containment in HIV-TB Co-Infection. PLoS.ONE. 2016; 11:e0146905 doi: 10.1371/journal.pone.0146905 26756579 10.1371/journal.pone.0146905 PMC4710462

[8] Houben RM, Crampin AC, Ndhlovu R, Sonnenberg P, Godfrey-Faussett P, Haas WH, et al Human immunodeficiency virus associated tuberculosis more often due to recent infection than reactivation of latent infection. Int.J.Tuberc.Lung Dis. 2011; 15:24–31. 21276292

[9] Goletti D, Sanduzzi A, Delogu G. Performance of the tuberculin skin test and interferon-gamma release assays: an update on the accuracy, cutoff stratification, and new potential immune-based approaches. J.Rheumatol.Suppl 2014; 91:24–31. doi: 10.3899/jrheum.140099 24788997 10.3899/jrheum.140099

[10] Goletti D, Carrara S, Mayanja-Kizza H, Baseke J, Mugerwa MA, Girardi E, et al Response to M. tuberculosis selected RD1 peptides in Ugandan HIV-infected patients with smear positive pulmonary tuberculosis: a pilot study. BMC.Infect.Dis. 2008; 8:11 doi: 10.1186/1471-2334-8-11 18226199 10.1186/1471-2334-8-11 PMC2267196

[11] Vincenti D, Carrara S, Butera O, Bizzoni F, Casetti R, Girardi E, et al Response to region of difference 1 (RD1) epitopes in human immunodeficiency virus (HIV)-infected individuals enrolled with suspected active tuberculosis: a pilot study. Clin.Exp.Immunol. 2007; 150:91–8. doi: 10.1111/j.1365-2249.2007.03462.x 17680823 10.1111/j.1365-2249.2007.03462.x PMC2219274

[12] Sester M, van Leth F, Bruchfeld J, Bumbacea D, Cirillo DM, Dilektasli AG, et al Risk assessment of tuberculosis in immunocompromised patients. A TBNET study. Am.J.Respir.Crit Care Med. 2014; 190:1168–76. doi: 10.1164/rccm.201405-0967OC 25303140 10.1164/rccm.201405-0967OC

[13] Launois P, Drowart A, Bourreau E, Couppie P, Farber CM, Van Vooren JP, et al T cell reactivity against mycolyl transferase antigen 85 of M. tuberculosis in HIV-TB coinfected subjects and in AIDS patients suffering from tuberculosis and nontuberculous mycobacterial infections. Clin.Dev.Immunol. 2011; 2011. 10.1155/2011/640309 PMC2948887 20936150

[14] Petruccioli E, Scriba TJ, Petrone L, Hatherill M, Cirillo DM, Joosten SA, et al Correlates of tuberculosis risk: predictive biomarkers for progression to active tuberculosis. Eur.Respir.J. 2016; 48:1751–63. doi: 10.1183/13993003.01012-2016 27836953 10.1183/13993003.01012-2016 PMC5898936

[15] Vanini V, Petruccioli E, Gioia C, Cuzzi G, Orchi N, Rianda A, et al IP-10 is an additional marker for tuberculosis (TB) detection in HIV-infected persons in a low-TB endemic country. J.Infect. 2012; 65:49–59. doi: 10.1016/j.jinf.2012.03.017 22465752 10.1016/j.jinf.2012.03.017

[16] Masungi C, Temmerman S, Van Vooren JP, Drowart A, Pethe K, Menozzi FD et al Differential T and B cell responses against Mycobacterium tuberculosis heparin-binding hemagglutinin adhesin in infected healthy individuals and patients with tuberculosis. J.Infect.Dis. 2002; 185:513–20. doi: 10.1086/338833 11865404 10.1086/338833

[17] Temmerman S, Pethe K, Parra M, Alonso S, Rouanet C, Pickett T, et al Methylation-dependent T cell immunity to Mycobacterium tuberculosis heparin-binding hemagglutinin. Nat.Med. 2004; 10:935–41. doi: 10.1038/nm1090 15300244 10.1038/nm1090

[18] Zanetti S, Bua A, Delogu G, Pusceddu C, Mura M, Saba F, et al Patients with pulmonary tuberculosis develop a strong humoral response against methylated heparin-binding hemagglutinin. Clin.Diagn.Lab Immunol. 2005; 12:1135–8. doi: 10.1128/CDLI.12.9.1135-1138.2005 16148186 10.1128/CDLI.12.9.1135-1138.2005 PMC1235805

[19] Delogu G, Chiacchio T, Vanini V, Butera O, Cuzzi G, Bua A, et al Methylated HBHA produced in M. smegmatis Discriminates between Active and Non-Active Tuberculosis Disease among RD1-Responders. PLoS.ONE. 2011; 6:e1815. 10.1371/journal.pone.0018315 PMC3066236 21479248

[20] Belay M, Legesse M, Mihret A, Ottenhoff TH, Franken KL, Bjune G, et al IFN-gamma and IgA against non-methylated heparin-binding hemagglutinin as markers of protective immunity and latent tuberculosis: Results of a longitudinal study from an endemic setting. J.Infect. 2016; 72:189–200. doi: 10.1016/j.jinf.2015.09.040 26518056 10.1016/j.jinf.2015.09.040

[21] Delogu G, Vanini V, Cuzzi G, Chiacchio T, De Maio F, Battah B, et al Lack of Response to HBHA in HIV-Infected Patients with Latent Tuberculosis Infection. Scand.J.Immunol. 2016; 84:344–52. doi: 10.1111/sji.12493 27636597 10.1111/sji.12493

[22] Delogu G., Bua A, Pusceddu C, Parra M, Fadda G, Brennan MJ, et al Expression and Purification of Recombinant Methylated HBHA in Mycobacterium smegmatis. FEMS Microbiol.Lett. 2004; 239:33–9. doi: 10.1016/j.femsle.2004.08.015 15451098 10.1016/j.femsle.2004.08.015

[23] Temmerman ST, Place S, Debrie AS, Locht C, Mascart F. Effector functions of heparin-binding hemagglutinin-specific CD8+ T lymphocytes in latent human tuberculosis. J.Infect.Dis. 2005; 192:226–32. doi: 10.1086/430930 15962217 10.1086/430930

[24] Goletti D, Parracino MP, Butera O, Bizzoni F, Casetti R, Dainotto D, et al Isoniazid prophylaxis differently modulates T-cell responses to RD1-epitopes in contacts recently exposed to Mycobacterium tuberculosis: a pilot study. Respir.Res. 2007; 8:5 doi: 10.1186/1465-9921-8-5 17257436 10.1186/1465-9921-8-5 PMC1794408

[25] Goletti D, Raja A, Syed Ahamed KB, Rodrigues C, Sodha A, Carrara S, et al Is IP-10 an accurate marker for detecting M. tuberculosis-specific response in HIV-infected persons? PLoS.ONE. 2010; 5:e12577 doi: 10.1371/journal.pone.0012577 20830287 10.1371/journal.pone.0012577 PMC2935361

[26] Petruccioli E, Petrone L, Vanini V, Sampaolesi A, Gualano G, Girardi E, et al IFNgamma/TNFalpha specific-cells and effector memory phenotype associate with active tuberculosis. J.Infect. 2013; 66:475–86. doi: 10.1016/j.jinf.2013.02.004 23462597 10.1016/j.jinf.2013.02.004

[27] Chiacchio T, Petruccioli E, Vanini V, Cuzzi G, Pinnetti C, Sampaolesi A, et al Polyfunctional T-cells and effector memory phenotype are associated with active TB in HIV-infected patients. J.Infect. 2014; 69:533–45. doi: 10.1016/j.jinf.2014.06.009 24975174 10.1016/j.jinf.2014.06.009

[28] Wyndham-Thomas C, Corbiere V, Dirix V, Smits K, Domont F, Libin M, et al Key role of effector memory CD4+ T lymphocytes in a short-incubation heparin-binding hemagglutinin gamma interferon release assay for the detection of latent tuberculosis. Clin.Vaccine Immunol. 2014; 21:321–8. doi: 10.1128/CVI.00651-13 24391135 10.1128/CVI.00651-13 PMC3957667

[29] Mascart F, Locht C. Integrating knowledge of Mycobacterium tuberculosis pathogenesis for the design of better vaccines. Expert.Rev.Vaccines. 2015; 14:1573–85. doi: 10.1586/14760584.2015.1102638 26517361 10.1586/14760584.2015.1102638

[30] Smits K, Corbiere V, Dirix V, Mekkaoui L, Wyndham-Thomas C, Libin M, et al Immunological signatures identifying different stages of latent Mycobacterium tuberculosis infection and discriminating latent from active tuberculosis in humans. Journal of Clinical & Cellular Immunology 2015; 6:341.

[31] Flynn JL, Chan J, Triebold KJ, Dalton DK, Stewart TA, Bloom BR. An essential role for interferon gamma in resistance to Mycobacterium tuberculosis infection. J.Exp.Med. 1993; 178:2249–54. 7504064 10.1084/jem.178.6.2249 PMC2191274

[32] Harari A, Rozot V, Enders FB, Perreau M, Stalder JM, Nicod LP, et al Dominant TNF-alpha(+) Mycobacterium tuberculosis-specific CD4(+) T cell responses discriminate between latent infection and active disease. Nat.Med. 2011; 17:372–6. doi: 10.1038/nm.2299 21336285 10.1038/nm.2299 PMC6570988

[33] Day CL, Abrahams DA, Lerumo L, Janse van Rensburg E, Stone L, O'rie T, et al Functional capacity of Mycobacterium tuberculosis-specific T cell responses in humans is associated with mycobacterial load. J.Immunol. 2011; 187:2222–32. doi: 10.4049/jimmunol.1101122 21775682 10.4049/jimmunol.1101122 PMC3159795

[34] Caccamo N, Guggino G, Joosten SA, Gelsomino G, Di Carlo P, Titone L, et al Multifunctional CD4(+) T-cells correlate with active Mycobacterium tuberculosis infection. Eur.J.Immunol. 2010; 40:2211–20. doi: 10.1002/eji.201040455 20540114 10.1002/eji.201040455

[35] Wilkinson KA, Wilkinson RJ. Polyfunctional T-cells in human tuberculosis. Eur.J.Immunol. 2010; 40:2139–42. doi: 10.1002/eji.201040731 20853500 10.1002/eji.201040731

[36] Tameris MD, Hatherill M, Landry BS, Scriba TJ, Snowden MA, Lockhart S, et al MVA85A 020 Trial Study Team. Safety and efficacy of MVA85A, a new tuberculosis vaccine, in infants previously vaccinated with BCG: a randomised, placebo-controlled phase 2b trial. Lancet. 2013;381:1021–8. doi: 10.1016/S0140-6736(13)60177-4 23391465 10.1016/S0140-6736(13)60177-4 PMC5424647

[37] Day CL, Mkhwanazi N, Reddy S, Mncube Z, van der Stok M, Klenerman P, et al Detection of polyfunctional Mycobacterium tuberculosis-specific T-cells and association with viral load in HIV-1-infected persons. J.Infect.Dis. 2008; 197:990–9. doi: 10.1086/529048 18419535 10.1086/529048 PMC5688849

[38] Pollock KM, Whitworth HS, Montamat-Sicotte DJ, Grass L, Cooke GS, Kapembwa MS, et al T-cell immunophenotyping distinguishes active from latent tuberculosis. J.Infect.Dis. 2013; 208:952–68. doi: 10.1093/infdis/jit265 23966657 10.1093/infdis/jit265 PMC3749005

[39] Beveridge NE, Fletcher HA, Hughes J, Pathan AA, Scriba TJ, Minassian A, et al A comparison of IFNgamma detection methods used in tuberculosis vaccine trials. Tuberculosis.(Edinb.) 2008; 88:631–40. 18801705 10.1016/j.tube.2008.06.005

[40] Parra M, Pickett T, Delogu G, Dheenadhayalan V, Debrie AS, Locht C, et al The Mycobacterial Heparin-binding Hemagglutinin is a Protective antigen in the Mouse Aerosol Challenge Model of Tuberculosis. Infect.Immun. 2004; 72:6799–805. doi: 10.1128/IAI.72.12.6799-6805.2004 15557600 10.1128/IAI.72.12.6799-6805.2004 PMC529156

[41] Stylianou E, Diogo GR, Pepponi I, van Dolleweerd C, Arias MA, Locht C, et al Mucosal delivery of antigen-coated nanoparticles to lungs confers protective immunity against tuberculosis infection in mice. Eur.J.Immunol. 2014; 44:440–9. doi: 10.1002/eji.201343887 24214530 10.1002/eji.201343887

[42] Wyndham-Thomas C, Dirix V, Schepers K, Martin C, Hildebrand M, Goffard JC, et al Contribution of a heparin-binding haemagglutinin interferon-gamma release assay to the detection of Mycobacterium tuberculosis infection in HIV-infected patients: comparison with the tuberculin skin test and the QuantiFERON-TB Gold In-tube. BMC.Infect.Dis. 2015; 15:59 doi: 10.1186/s12879-015-0796-0 25886172 10.1186/s12879-015-0796-0 PMC4337251

[43] Lewinsohn DA, Heinzel AS, Gardner JM, Zhu L, Alderson MR, Lewinsohn DM. Mycobacterium tuberculosis-specific CD8+ T-cells preferentially recognize heavily infected cells. Am.J.Respir.Crit Care Med. 2003; 168:1346–52. doi: 10.1164/rccm.200306-837OC 12969871 10.1164/rccm.200306-837OC

[44] Stenger S, Hanson DA, Teitelbaum R, Dewan P, Niazi KR, Froelich CJ, et al An antimicrobial activity of cytolytic T-cells mediated by granulysin. Science 1998; 282:121–5. 9756476 10.1126/science.282.5386.121

[45] Petruccioli E, Chiacchio T, Pepponi I, Vanini V, Urso R, Cuzzi G, et al First characterization of the CD4 and CD8 T-cell responses to QuantiFERON-TB Plus. J.Infect. 2016; 73:588–97. doi: 10.1016/j.jinf.2016.09.008 27717779 10.1016/j.jinf.2016.09.008

[46] Getahun H, Matteelli A, Abubakar I, Aziz MA, Baddeley A, Barreira D, et al Management of latent Mycobacterium tuberculosis infection: WHO guidelines for low tuberculosis burden countries. Eur.Respir.J. 2015; 46:1563–76. doi: 10.1183/13993003.01245-2015 26405286 10.1183/13993003.01245-2015 PMC4664608

[47] Corbiere V, Pottier G, Bonkain F, Schepers K, Verscheure V, Lecher S, et al Risk stratification of latent tuberculosis defined by combined interferon gamma release assays. PLoS.ONE. 2012; 7:e43285 doi: 10.1371/journal.pone.0043285 22912846 10.1371/journal.pone.0043285 PMC3422279

[48] Mack U, Migliori GB, Sester M, Rieder HL, Ehlers S, Goletti D, et al LTBI: latent tuberculosis infection or lasting immune responses to M. tuberculosis? A TBNET consensus statement. Eur.Respir.J. 2009; 33:956–73. doi: 10.1183/09031936.00120908 19407047 10.1183/09031936.00120908

[49] Sester M, Sotgiu G, Lange C, Giehl C, Girardi E, Migliori GB, et al Interferon-gamma release assays for the diagnosis of active tuberculosis: a systematic review and meta-analysis. Eur.Respir.J. 2011; 37:100–11. doi: 10.1183/09031936.00114810 20847080 10.1183/09031936.00114810

[50] Petruccioli E, Vanini V, Chiacchio T, Cuzzi G, Cirillo DM, Palmieri F, et al Analytical evaluation of QuantiFERON-Plus and QuantiFERON-Gold In-tube assays in subjects with or without tuberculosis. Tuberculosis. 2017 9;106:38–43. doi: 10.1016/j.tube.2017.06.002 28802403 10.1016/j.tube.2017.06.002

