# Supplementary figures and images for: Immune characterization of the HBHA-specific response in Mycobacterium tuberculosis-infected patients with or without HIV infection
Source: PLoS One. 2017 Aug 24;12(8):e0183846. doi: 10.1371/journal.pone.0183846 (PMC5570327; doi:10.1371/journal.pone.0183846)

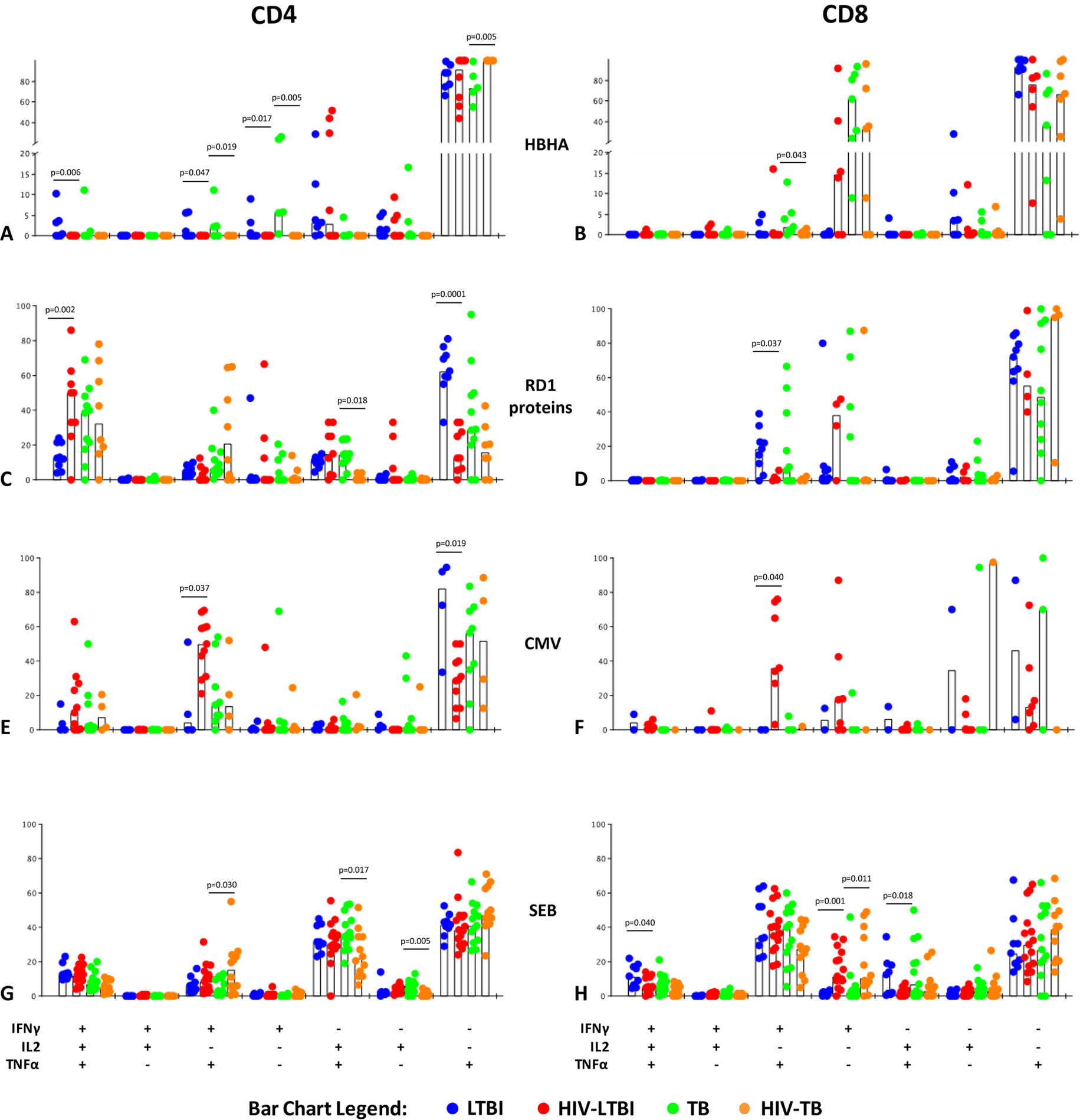

Supplement: S1 Fig — PBMC were stimulated overnight with HBHA (A, B), RD1 proteins (C, D), CMV (E, F) and SEB (G, H) and analyzed by flow cytometry for intracellular production of IFNγ, TNFα and IL2. A-H: The bar graphs show the proportion of stimuli cytokine producing CD4+ and CD8+ T-cells over the total responding T-cells in the different groups analyzed. The horizontal lines represent the median; blue circles represent the LTBI group, red circles represent the HIV-LTBI group; green circles represent the TB group; orange circles represent the HIV-TB group. Statistical analysis was performed using Mann-Whitney test and p value was considered significant if < 0.05. A, C, E, G) Proportion of cytokine-producing CD4+ T-cells in response to each stimulus; B, D, F, H) Proportion of cytokine-producing CD8+ T-cells in response to each stimulus. Footnotes: HIV: human immunodeficiency virus; TB: tuberculosis; LTBI: latent TB infection; HBHA: heparin-binding haemagglutinin; RD: region of difference; CMV: cytomegalovirus; SEB: staphylococcal enterotoxin B; IFN: interferon; IL: interleukin; TNF: tumor necrosis factor. (TIF) [file pone.0183846.s001.tif]

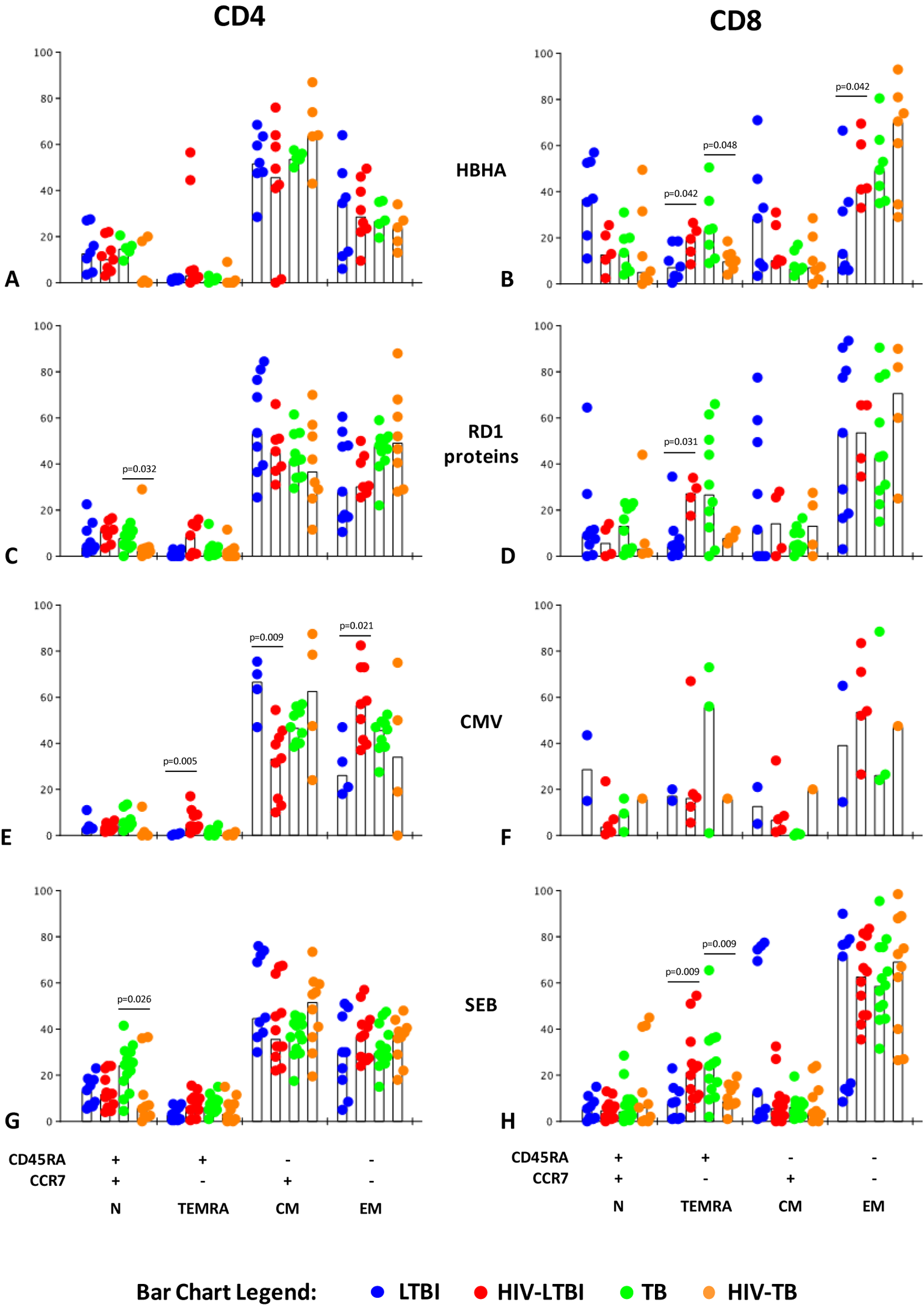

Supplement: S2 Fig — Memory status of CD4+ and CD8+ T-cell response was evaluated by flow cytometry according to the surface expression of CD45RA and CCR7 in the gate of total CD4 and CD8 T-cell response. We defined naïve (N) as CD45RA+ CCR7+, terminally differentiated effector memory T-cells (TEMRA) as CD45RA+ CCR7-, central memory (CM) as CD45RA- CCR7+ and effector memory (EM) as CD45RA- CCR7-. A-H: The bar graphs represent the proportion of N, TEMRA, CM, and EM CD4+ and CD8+ T-cells in the different groups analyzed in response to overnight stimulation with the different stimuli. The horizontal lines represent the median; blue circles represent the LTBI group, red circles represent the HIV-LTBI group; green circles represent the TB group; orange circles represent the HIV-TB group. Statistical analysis was performed using Mann-Whitney test and p value was considered significant if < 0.05. A, C, E, G) Phenotype of CD4+ T-cells in response to each stimulus; B, D, F, H) Phenotype of CD8 T-cells in response to each stimulus. Footnotes: HIV: human immunodeficiency virus; TB: tuberculosis; LTBI: latent TB infection; HBHA: heparin-binding haemagglutinin; RD: region of difference; CMV: cytomegalovirus; SEB: staphylococcal enterotoxin B; N: naïve; TEMRA: terminally-differentiated effector memory; CM: central memory; EM effector memory. (TIF) [file pone.0183846.s002.tif]
